# Characterization of the complete mitochondrial genomes of *Nematodirus oiratianus* and *Nematodirus spathiger* of small ruminants

**DOI:** 10.1186/1756-3305-7-319

**Published:** 2014-07-11

**Authors:** Guang-Hui Zhao, Yan-Qing Jia, Wen-Yu Cheng, Wen Zhao, Qing-Qing Bian, Guo-Hua Liu

**Affiliations:** 1 College of Veterinary Medicine, Northwest A&F University, Yangling, Shaanxi Province 712100, People’s Republic of China; 2 Lanzhou Veterinary Research Institute, State Key Laboratory of Veterinary Etiological Biology, Key Laboratory of Veterinary Parasitology of Gansu Province, Chinese Academy of Agricultural Sciences, Lanzhou, Gansu Province 730046, People’s Republic of China

**Keywords:** *Nematodirus oiratianus*, *Nematodirus spathiger*, Mitochondrial genome, Phylogenetic analyses

## Abstract

**Background:**

*Nematodirus* spp. are among the most common nematodes of ruminants worldwide. *N. oiratianus* and *N. spathiger* are distributed worldwide as highly prevalent gastrointestinal nematodes, which cause emerging health problems and economic losses. Accurate identification of *Nematodirus* species is essential to develop effective control strategies for *Nematodirus* infection in ruminants. Mitochondrial DNA (mtDNA) could provide powerful genetic markers for identifying these closely related species and resolving phylogenetic relationships at different taxonomic levels.

**Methods:**

In the present study, the complete mitochondrial (mt) genomes of *N. oiratianus* and *N. spathiger* from small ruminants in China were obtained using Long-range PCR and sequencing.

**Results:**

The complete mt genomes of *N. oiratianus* and *N. spathiger* were 13,765 bp and 13,519 bp in length, respectively. Both mt genomes were circular and consisted of 36 genes, including 12 genes encoding proteins, 2 genes encoding rRNA, and 22 genes encoding tRNA. Phylogenetic analyses based on the concatenated amino acid sequence data of all 12 protein-coding genes by Bayesian inference (BI), Maximum likelihood (ML) and Maximum parsimony (MP) showed that the two *Nematodirus* species (Molineidae) were closely related to Dictyocaulidae.

**Conclusions:**

The availability of the complete mtDNA sequences of *N. oiratianus* and *N. spathiger* not only provides new mtDNA sources for a better understanding of nematode mt genomics and phylogeny, but also provides novel and useful genetic markers for studying diagnosis, population genetics and molecular epidemiology of *Nematodirus* spp. in small ruminants.

## Background

*Nematodirus* spp. are among the most common nematodes of ruminants and more than 45 species have been described in the genus *Nematodirus* Ransom, 1907 [1,2]. Of these, *Nematodirus oiratianus* and *N. spathiger* are widely distributed as highly prevalent gastrointestinal nematodes, mainly inhabiting the small intestines of sheep and goats [3-5]. Although mild, or no symptoms are usually presented during *Nematodirus* infection in adult ruminants, growth retardation and emaciation of the growing host during infection may result in economic losses. During winter, *Nematodirus* eggs are able to develop to the gastrula stage within two weeks and then to infective larvae after another 4 weeks, and are strongly resistant to adverse environmental conditions [6]. These larvae therefore represent a potent source of infection during spring leading to high numbers of infected ruminants early in the growing season [6,7].

Traditionally, *Nematodirus* species have been identified solely on morphological features of the adults, including characteristics of the spicule tips and copulatory bursae [8]. However, such criteria are often insufficient for specific identification and differentiation of *Nematodirus* species, especially for eggs, larvae and females [9,10]. Due to the limitations of morphological approaches, various molecular methods have been used widely for the identification and differentiation of *Nematodirus* species. The internal transcribed spacer (ITS) of nuclear ribosomal DNA (rDNA) has previously been described as a useful marker for the identification and differentiation of Molineid nematodes [11-13]. At least eight *Nematodirus* species, including *N. battus*, *N. davtiani alpinus*, *N. europaeus*, *N. filicollis*, *N. helvetianus*, *N. oiratianus*, *N. spathiger* and *N. rupicaprae*, could be identified by their ITS rDNA sequences [11-13]. Compared to nuclear rDNA, mitochondrial (mt) DNA (mtDNA) is more reliable for identifying closely related species, particularly cryptic species, since the mtDNA sequences accumulate nucleotide substitutions much more quickly than ITS rDNA [14]. Indeed, various studies have indicated that mt genome sequences provide powerful genetic markers in resolving phylogenetic relationships at different taxonomic levels, particularly when concatenated protein-coding sequences are used for phylogenetic analysis [15-22].

Based on recent progress in Long-range PCR-coupled sequencing and bioinformatic methods [23,24], the objectives of the present study were to sequence and compare the complete mt genomes of *N. oiratianus* and *N. spathiger*. We also assessed phylogenetic relationships of the two *Nematodirus* species with a range of other Trichostrongyloid nematodes using complete, inferred mt protein sequence data sets.

## Methods

### Ethics statement

The performance of this study was strictly according to the recommendations of the Guide for the Care and Use of Laboratory Animals of the Ministry of Health, China, and our protocol was reviewed and approved by the Research Ethics Committee of Northwest A&F University.

### Parasites

All the nematode samples were collected from animals with the permission of the Laboratory of Veterinary Parasitology of Northwest A&F University, with no specific permits being required by the authority for the sample collection.

### Genomic DNA extraction

Female adults of *N. oiratianus* (Code: YLF2) and *N. spathiger* (JYF2) were obtained from the small intestines of naturally-infected goats and sheep, respectively, in Shaanxi Province, China. Adult worms from each host were washed separately in physiological saline, fixed in 70% (v/v) ethanol and stored at -20°C until further study. *Nematodirus* species were firstly identified morphologically according to characteristics of the spicule tips and copulatory bursae and distribution of bosses on the internal surfaces of the bursae [5,8]. For each species, individual males were identified on the basis of bursal form, number of cuticular ridges and spicule morphology [25].

The *Nematodirus* species were further identified using a molecular method based on ITS rDNA. Total genomic DNA was isolated separately from individual worms of each species by proteinase K treatment, column-purification (TIANamp Genomic DNA Purification System, TIANGEN, China) and elution into 40 μl H_2_O according to the manufacturer’s recommendations. The region spanning ITS-1, 5.8S and ITS-2 rDNA was amplified from each individual using universal primers NC5 and NC2 [26] and sequenced directly. Phylogenetic analyses based on the ITS-2 rDNA sequences, using Maximum parsimony (MP) and Maximum likelihood (ML) methods, were used to further determine the *Nematodirus* species used in this study.

### Long-range PCR-based sequencing of mtDNA

Using primers designed against relatively conserved regions within the *cox*1, *rrn*L and *nad*1 regions (Additional file 1) [27], the complete mt genome was amplified from total genomic DNA (from an individual worm) as four overlapping fragments between *rrn*L and *nad*1, *nad*1, *nad*1 and *cox*1, and *cox*1 and *rrn*L (Additional file 1), respectively. Each fragment was amplified by long-range PCR using LA TAQ polymerase (TAKARA, China), following the manufacturer’s recommendations. The cycling conditions used were 92°C for 2 min (initial denaturation), then 92°C for 10 s (denaturation), 45°C for 30 s (annealing), and 60°C for 8 min (extension) for 9 cycles, followed by 92°C for 10 s, 45°C for 30 s, and 60°C for 9 min for 25 cycles, and a final extension at 60°C for 10 min. Each amplicon was represented by a single band in a 1.0% (w/v) agarose gel, following electrophoresis and ethidium-bromide staining. The amplicon was column-purified and then sequenced using a primer walking strategy [23].

### Sequence annotation

Sequences were assembled manually and aligned against the complete mt genome sequences of other nematodes (available in GenBank) using the computer program MAFFT 7 [28] to infer gene boundaries. The open-reading frames (ORFs) were predicted by the Open Reading Frame Finder (http://www.ncbi.nlm.nih.gov/gorf/gorf.html) using the invertebrate mitochondrial code and subsequently compared with those of *Trichostrongylus axei* and *Trichostrongylus vitrinus*[29]. Each gene was translated into amino acid sequence using the invertebrate mitochondrial genetic code in MEGA 5 [30], and aligned based on its amino acid sequence using default settings. The alignment was back-translated into the corresponding nucleotide sequences. The translation initiation and termination codons were identified to avoid gene overlap and to optimize the similarity between the gene lengths of closely-related nematode mitochondrial genomes. Codon usages were examined based on the relationships between the nucleotide composition of codon families and amino acid occurrence, for which codons are partitioned into AT-rich codons, GC-rich codons and unbiased codons. The secondary structures of 22 tRNA genes were predicted using tRNAscan-SE [31] and/or manual adjustment [32], and the rRNA genes were predicted by comparison with those of closely-related nematodes (*T. axei* and *T. vitrinus*[29]) and their secondary structures.

### Phylogenetic analyses

Amino acid sequences inferred from the 12 protein-coding genes were concatenated into a single alignment, and then aligned with those of eight other Trichostrongyloid nematodes (*Cooperia oncophora*, NC_004806 [33]; *Haemonchus contortus*, NC_010383 [24]; *T. axei*, NC_013824 [29]; *T. vitrinus*, NC_013807 [29]; *Teladorsagia circumcincta*, NC_013827 [29]; *Mecistocirrus digitatus*, NC_013848 [29]; *Dictyocaulus viviparus*, NC_019810 [34]; *Dictyocaulus eckerti*, NC_019809) [34]), using the Strongyloid nematode *Oesophagostomum quadrispinulatum* (NC_014181) as the outgroup [35]. Any regions of ambiguous alignment were excluded using Gblocks online server [36] (http://molevol.cmima.csic.es/castresana/Gblocks_server.html) using the options for less stringent selection. Phylogenetic analyses were conducted using three methods: Bayesian inference (BI), Maximum parsimony (MP) and Maximum likelihood (ML). The MtArt + G + F model of amino acid evolution was selected as the most suitable model of evolution by ProtTest 2.4 [37] based on the Akaike information criterion (AIC). As the MtArt model is not implemented in the current version of MrBayes, an alternative model, MtREV, was used in BI and four chains (three heated and one cold) were run simultaneously for the Monte Carlo Markov Chain. Two independent runs were performed for 1,000,000 metropolis-coupled MCMC generations, sampling a tree every 100 generation in MrBayes 3.1.1 [38]; the first 2,500 trees represented burn-in and the remaining trees were used to calculate Bayesian posterior probabilities (Bpp). MP analysis was conducted using PAUP 4.0 Beta 10 program [39], with indels treated as missing character states; 1,000 random additional searches were performed using TBR. Bootstrap frequency (Bf) was calculated using 1,000 bootstrap replicates, and 100 random taxon additions in PAUP. ML analysis was performed using PhyML 3.0 [40]. Bf was calculated using 100 bootstrap replicates. Phylograms were drawn using the program Tree View v.1.65 [41].

## Results and discussion

Both nematode species have cephalic vesicles, two sets of reproductive organs, a single distribution of large black eggs, and a conical end with a transparent thin spine. Usually, species identification mostly depends on male morphological features, but in this study, identification of the parasites was mainly dependent on ITS rDNA sequences as all worms used herein were female [8,42]. The region spanning ITS-1, 5.8S and ITS-2 rDNA was amplified from each individual worm using universal primers NC5 and NC2 [26] and sequenced directly. The ITS-1 rDNA sequences (GenBank accession nos. KC580735 and KC580745, respectively) of the *N. oiratianus* and *N. spathiger* samples had 96% and 99% identity to those of *N. oiratianus* and *N. spathiger* in public databases (HQ389233 and AF194144, respectively). The ITS-2 rDNA sequences (KC580735 and KC580745, respectively) of the *N. oiratianus* and *N. spathiger* samples had 94% and 100% identity to those of *N. oiratianus* and *N. spathiger* in public databases (HQ389233 and KC998746, respectively) [2]. Phylogenetic analysis based on the ITS-2 rDNA sequences, using Maximum parsimony (MP) and Maximum likelihood (ML), further showed that the *Nematodirus* isolates represented *N. oiratianus* and *N. spathiger*, respectively (Additional file 2).

**Figure 1:**
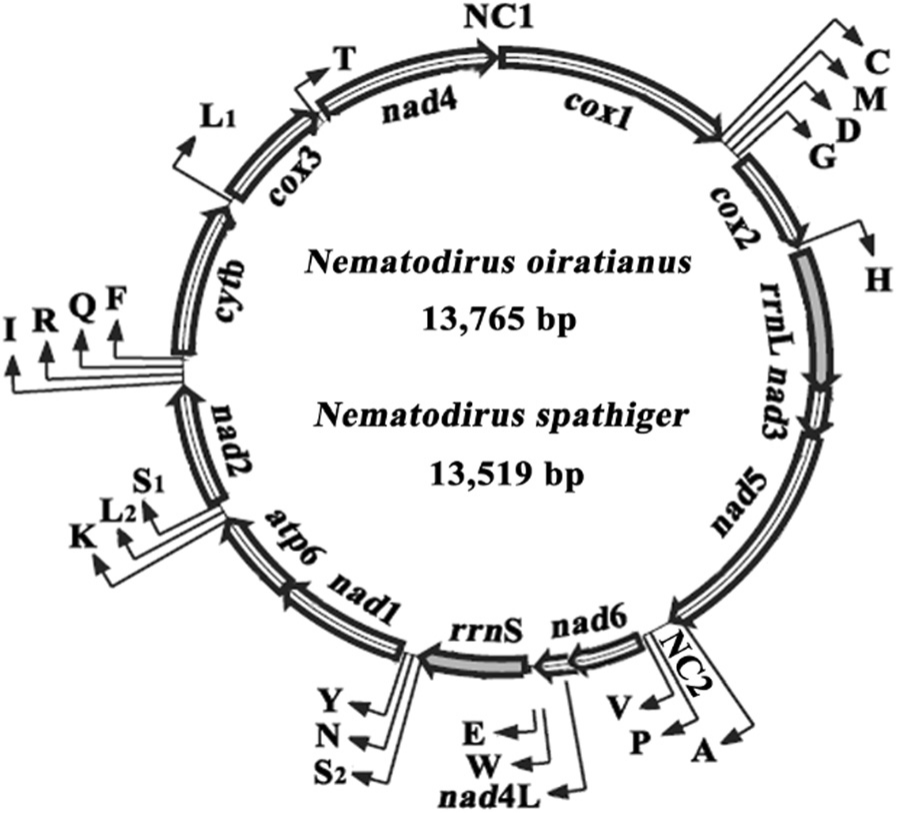
**Structure of the mitochondrial genomes of *Nematodirus oiratianus *and *Nematodirus spathiger.*** Genes are designated according to standard nomenclature, except for the 22 tRNA genes, which are designated using one-letter amino acid codes, with numerals differentiating each of the two leucine- and serine-specifying tRNAs (L1 and L2 for codon families CUN and UUR, respectively; S1 and S2 for codon families AGN and UCN, respectively). “NC1” refers to a small non-coding region; “NC2” refers to a large non-coding region.

The respective lengths of the four overlapping fragments amplified by long-PCR covering the entire mt genomes of *N. oiratianus* and *N. spathiger* were 4765 bp and 4741 bp for *rrn*L-*nad*1, 511 bp and 510 bp for partial *nad*1, 7084 bp and 7060 bp for *nad*1-*cox*1, and 2330 bp and 2320 bp for *cox*1-*rrn*L, respectively. After sequence splicing, the complete mt genomes of *N. oiratianus* and *N. spathiger* (KF573750 and KF573749) were 13,765 bp and 13,519 bp in length, respectively (Figure 1). Both mt genomes contained 12 protein-coding genes (*cox*1-3, *nad*1-6, *nad*4L, *atp*6 and *cyt*b), 22 transfer RNA genes, two ribosomal RNA genes and two non-coding regions, but lacked an *atp*8 gene (Table 1). These circular genomes are typical mt genomes of Chromadorea nematodes, such as *Ascaris suum*[15], *Enterobius vermicularis*[43], *Oesophagostomum* spp*.*[44] and *Spirocerca lupi*[45]. All mt genes were transcribed in the same direction, with same gene order as Gene arrangement 3 (GA3): *nad*6 > *nad*4L > tRNA-Trp (W) > tRNA-Glu (E) > *rrn*S > tRNA-SerUCN (S2) > tRNA-Asn (N) > tRNA-Tyr (Y) > *nad*1 > *atp*6 > tRNA-Lys (K) > tRNA-LeuUUR (L2) > tRNA-SerAGN (S1) > *nad*2 > tRNA-Ile (I) > tRNA-Arg (R) > tRNA-Gln (Q) > tRNA-Phe (F) > *cyt*b > tRNA-LeuCUN (L1) > *cox*3 > tRNA-Thr (T) > *nad*4 > Non-coding region (NC1) > *cox*1 > tRNA-Cys (C) > tRNA-Met (M) > tRNA-Asp (D) > tRNA-Gly (G) > *cox*2 > tRNA-His (H) > *rrn*L > *nad*3 > *nad*5 > tRNA-Ala (A) > Non-coding region (NC2) > tRNA-Pro (P) > tRNA-Val (V) (Figure 1, Table 1).The nucleotide compositions of the completemt DNA sequences for *N. oiratianus* and *N. spathiger* are biased toward T and A, with T being the most favored nucleotide and C as the least favored. The contents of A + T were 76.14% (T = 47.69%; A = 28.46%; G = 16.8%; C = 7.06%) and 75.04% (T = 48.29%; A = 26.75%; G = 17.69%; C = 7.27%) for *N. oiratianus* and *N. spathiger*, respectively. The magnitude of sequence difference across the complete mt genome was 16.29% between *N. oiratianus* and *N. spathiger*.

**Table 1:** **The arrangements and contents of mitochondrial genomes for *Nematodirus spathiger *** **and *N. oiratianus***

**Genes/regions**	**Positions**		**Length**		**Start/stop**		**Sequence difference (%)**
	**NO**	**NS**	**NO**	**NS**	**NO**	**NS**	
*nad*6	1-438	1-438	438	438	ATG /TAG	ATA /TAA	20.78
*nad*4L	447-695	441-689	249	249	ATT/TAG	ATT/TAA	12.05
tRNA-Trp (W)	676-731	670-725	56	56			5.36
tRNA-Glu (E)	739-793	731-785	55	55			10.91
*rrn*S	794-1489	787-1481	696	695			9.05
tRNA-SerUCN (S2)	1490-1543	1482-1534	54	53			9.26
tRNA-Asn (N)	1544-1599	1536-1589	56	54			10.71
tRNA-Tyr (Y)	1606-1659	1596-1651	54	56			5.36
*nad*1	1666-2532	1658-2524	867	867	ATG/TAA	ATA /TAA	12.34
*atp*6	2533-3132	2524-3123	600	600	ATT/TAA	ATT /TAA	14.17
tRNA-Lys (K)	3131-3193	3127-3187	63	61			12.7
tRNA-LeuUUR (L2)	3195-3249	3189-3243	55	55			0
tRNA-SerAGN (S1)	3250-3300	3244-3295	51	52			19.23
*nad*2	3307-4144	3302-4140	838	839	ATG/T	ATG/TA	8.34
tRNA-Ile (I)	4145-4200	4141-4196	56	56			19.64
tRNA-Arg (R)	4221-4274	4197-4251	54	55			3.64
tRNA-Gln (Q)	4328-4383	4254-4309	56	56			10.71
tRNA-Phe (F)	4396-4451	4316-4369	56	54			14.29
*cyt*b	4452-5564	4370-5481	1113	1112	ATT/TAA	ATT/TA	5.75
tRNA-LeuCUN (L1)	5565-5620	5482-5537	56	56			14.29
*cox*3	5621-6386	5538-6303	766	766	ATT/T	ATA/T	10.18
tRNA-Thr (T)	6387-6441	6304-6357	55	54			27.27
*nad*4	6442-7671	6358-7587	1230	1230	TTG/TAA	TTG/TAA	17.32
Non-coding region (NC1)	7672-7769	7588-7685	98	98			17.35
*cox*1	7770-9345	7686-9261	1576	1576	ATA/T	ATA/T	11.55
tRNA-Cys (C)	9346-9399	9262-9316	54	55			16.36
tRNA-Met (M)	9401-9456	9319-9373	56	55			7.14
tRNA-Asp (D)	9457-9511	9373-9428	55	56			12.5
tRNA-Gly (G)	9513-9567	9430-9485	55	56			12.5
*cox*2	9568-10261	9486-10181	694	696	ATT/T	ATT/TAG	12.79
tRNA-His (H)	10262-10315	10187-10240	54	54			20.37
*rrn*L	10316-11277	10241-11198	962	958			5.2
*nad*3	11278-11604	11199-11525	327	327	ATA/TAG	ATG/TAA	12.54
*nad*5	11608-13189	11525-13106	1582	1582	ATT/T	ATT/T	11.06
tRNA-Ala (A)	13190-13245	13107-13162	56	56			1.79
Non-coding region (NC2)	13246-13633	13163-13400	388	238			55.93
tRNA-Pro (P)	13634-13687	13401-13456	54	56			10.71
tRNA-Val (V)	13712-13765	13466-13519	54	54			5.56

NS: *N. spathiger*; NO: *N. oiratiuns*; Start/Stop: initiation and termination codons.

All protein-coding genes used ATA, ATT, ATG or TTG as their initiation codons, and TAA or TAG as their termination codons. Incomplete termination codons (TA and T) were also present (Table 1), which was consistent with studies of some other nematodes [15,16,44]. Excluding the termination codons, a total of 3419 and 3416 amino acids were encoded by the *N. oiratianus* and *N. spathiger* mt genomes, respectively. The codon usages for the two mt genomes are listed in Table 2. Codons composed of A and T were predominantly used, reflecting the high content of A + T in the complete mt genomes of the two *Nematodirus* species. The most frequent usage of codon was TTT (Phenylalanine) for both two species, with frequencies of 12.61% and 12.88% for *N. oiratianus* and *N. spathiger*, respectively, followed by TTA (Leucine) in the two species (10.95% and 9.17%, respectively). Codon CGC (Arginine) was not used in the mt genomes of either species, and codon TGC (Cysteine) was not used in the mt genome of *N. spathiger*.

**Table 2:** **Codon usages of *Nematodirus oiratianus *** **and *N. spathiger *** **mitochondrial DNA encoded proteins**

**Amino acid**	**Codon**	**Number**		**Frequency (%)**		**Amino acid**	**Codon**	**Number**		**Frequency (%)**	
		**NO**	**NS**	**NO**	**NS**			**NO**	**NS**	**NO**	**NS**
Phe	TTT	432	441	12.61	12.88	IIe	ATT	229	245	6.68	7.16
Phe	TTC	8	9	0.23	0.26	IIe	ATC	10	4	0.29	0.12
Leu	TTA	375	314	10.95	9.17	Met	ATA	150	125	4.38	3.65
Leu	TTG	160	194	4.67	5.67	Met	ATG	69	80	2.01	2.34
Ser	TCT	75	89	2.19	2.6	Thr	ACT	74	74	2.16	2.16
Ser	TCC	2	10	0.06	0.29	Thr	ACC	9	10	0.26	0.29
Ser	TCA	48	33	1.4	0.96	Thr	ACA	21	17	0.61	0.5
Ser	TCG	14	11	0.41	0.32	Thr	ACG	10	8	0.29	0.23
Tyr	TAT	182	190	5.31	5.55	Asn	AAT	135	130	3.94	3.8
Tyr	TAC	9	5	0.26	0.15	Asn	AAC	4	8	0.12	0.23
Term	TAA	5	6	0.15	0.18	Lys	AAA	49	41	1.43	1.2
Term	TAG	2	1	0.06	0.03	Lys	AAG	52	59	1.52	1.72
Cys	TGT	39	39	1.14	1.14	Ser	AGT	141	164	4.12	4.79
Cys	TGC	2	0	0.06	0	Ser	AGC	3	4	0.09	0.12
Trp	TGA	41	32	1.2	0.93	Ser	AGA	50	30	1.46	0.88
Trp	TGG	29	38	0.85	1.11	Ser	AGG	28	31	0.82	0.91
Leu	CTT	14	16	0.41	0.47	Val	GTT	141	146	4.12	4.27
Leu	CTC	4	3	0.12	0.09	Val	GTC	12	12	0.35	0.35
Leu	CTA	12	11	0.35	0.32	Val	GTA	94	76	2.74	2.22
Leu	CTG	5	11	0.15	0.32	Val	GTG	43	64	1.26	1.87
Pro	CCT	41	45	1.2	1.31	Ala	GCT	72	69	2.1	2.02
Pro	CCC	5	11	0.15	0.32	Ala	GCC	11	14	0.32	0.41
Pro	CCA	22	15	0.64	0.44	Ala	GCA	17	18	0.5	0.53
Pro	CCG	9	6	0.26	0.18	Ala	GCG	4	8	0.12	0.23
His	CAT	46	49	1.34	1.43	Asp	GAT	64	63	1.87	1.84
His	CAC	10	5	0.29	0.15	Asp	GAC	4	4	0.12	0.12
Gln	CAA	18	23	0.53	0.67	Glu	GAA	41	30	1.2	0.88
Gln	CAG	22	18	0.64	0.53	Glu	GAG	33	44	0.96	1.29
Arg	CGT	26	27	0.76	0.79	Gly	GGT	127	133	3.71	3.89
Arg	CGC	0	0	0	0	Gly	GGC	5	6	0.15	0.18
Arg	CGA	4	1	0.12	0.03	Gly	GGA	28	17	0.82	0.5
Arg	CGG	1	3	0.03	0.09	Gly	GGG	34	33	0.99	0.96

NO: *N. oiratiuns*; NS: *N. spathiger*; Total number of codon are 3426 for *N. oiratiuns*, and 3423 for *N. spathiger*, excluding the incomplete termination codons.

Twenty-two tRNA genes were predicted from the mt genomes of *N. oiratianus* and *N. spathiger* and varied from 51 to 63 bp in length. The secondary structures predicted for the genes were similar to those of *T. axei* and *T. vitrinus*[29]. Twenty tRNA genes (excluding two tRNA-Ser) had a predicted secondary structure with a 3-5 bp DHU arm and a DHU loop of 7-9 bases, in which the variable TψC arm and loop were replaced by a “TV-replacement loop” of 8-10 bases. As in almost all other nematode mtDNA sequences [29], the tRNA-Ser genes of both *Nematodirus* mt genomes are equipped with a TψC arm and loop but lacked the DHU arm and loop, consisting of a 6-8 bp TψC arm, TψC loop of 4-6 bases and a variable loop of 4 bases.

The *rrn*L and *rrn*S genes of *N. oiratianus* and *N. spathiger* were identified by comparison with those of *T. axei* and *T. vitrinus*[29]. The *rrn*L gene was located between tRNA-His and *nad*3, and *rrn*S was located between tRNA-Glu and tRNA-Ser^UCN^ (Table 1). The length of *rrn*L was 962 bp for *N. oiratianus* and 958 bp for *N. spathiger*, with 5.2% of sequence difference. The length of *rrn*S was 696 bp for *N. oiratianus* and 695 bp for *N. spathiger*, with 9.05% of sequence difference. The A + T contents of *rrn*L for *N. oiratianus* and *N. spathiger* were 80.98% and 79.85%, respectively. The A + T contents of *rrn*S for *N. oiratianus* and *N. spathiger* were 76.29% and 77.70%, respectively.

Two non-coding regions (designated as NC1 and NC2) were inferred in the mt genomes of both *N. oiratianus* and *N. spathiger*. For both mt genomes, the AT-rich regions were located between *nad*4 and *cox*1, tRNA-Ala and tRNA-Pro (Figure 1; Table 1), with A + T contents of 81.25% for *N. oiratianus* and 81.07% for *N. spathiger*.

**Figure 2:**
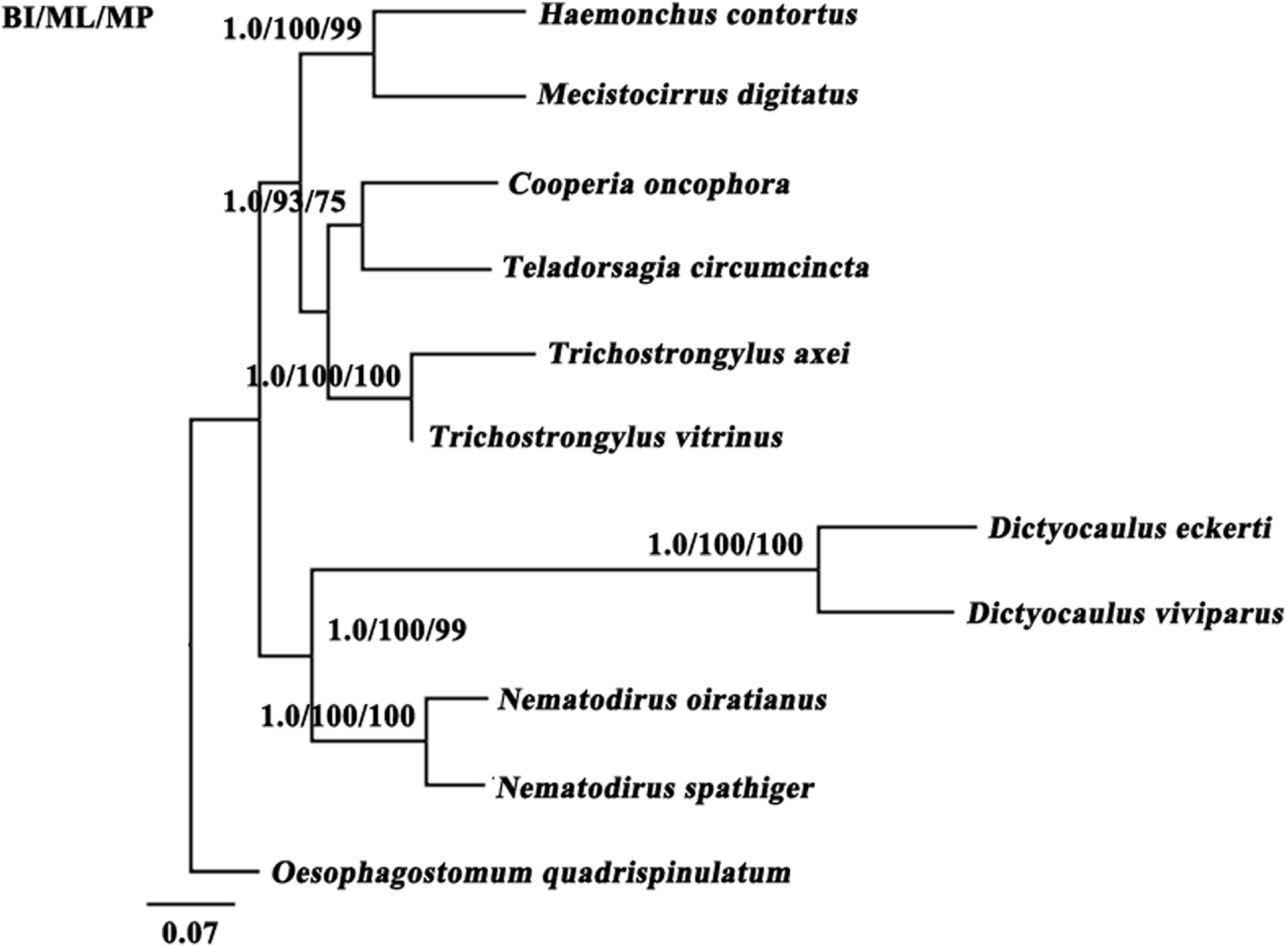
**Genetic relationships of *Nematodirus *nematodes with other selected Trichostrongyloid nematodes inferred by Bayesian inference (BI) based on mitochondrial sequence data.** Phylogenetic analyses based on the concatenated amino acid sequence data representing 12 protein-coding genes were conducted using Bayesian inference (BI), Maximum likelihood (ML) and Maximum parsimony (MP), with *Oesophagostomum quadrispinulatum* (NC_014181) as the outgroup. The scale bar indicated Posterior Probability.

Phylogenetic analysis (Figure 2) based on concatenated amino acid sequence data of all 12 mt proteins showed that nematodes in families Molineidae and Dictyocaulidae grouped together, and parasites in families Trichostrongylidae (*T. vitrinus*, *T. axei* and *T. circumcincta*), Cooperiidae (*Cooperia oncophora*) and Haemonchidae (*M. digitatus*and *H. contortus*) clustered together. *N. oiratianus* and *N. spathiger* samples in the present study clustered together and were highly closely related to the family Dictyocaulidae with high clade support (Bpp = 1.00; Bf = 100), confirming the results of previous studies using the morphological features and ITS rDNA as the genetic marker [2,9,42]. However, amino acid sequence-based inferences of the relationship between the two *Nematodirus* species gave slightly different results to those using ITS rDNA data in which they clustered in sister clades with high support, possibly due to the different taxa inclusions and types of analysis performed [2,11-13,46].

Molecular analyses have provided new insights into population structure and species identification of parasites [15,16,34,44]. In the present study, we utilized long-range PCR-coupled sequencing and bioinformatic methods [23] to characterize the complete mt genomes of two *Nematodirus* species from small ruminants and to assess the phylogenetic relationships of these *Nematodirus* species in relation to representative Trichostrongyloid nematodes. Given that there are no morphological characteristics to allow the explicit specific identification and differentiation of *Nematodirus* species (Nematoda: Molineidae) at some developmental stages, such as larvae and egg [9,10], the use of mt DNA markers to investigate genetic composition in these species is a significant advance. To date, more than 45 *Nematodirus* species have been described based on their morphological features [1,2], however, no information is yet available about their mt genomes. Although previous studies have indicated that ITS rDNA provides a useful marker for identification and differentiation of *Nematodirus* species [2,11,13], mtDNA in nematodes is usually more variable in sequences within a species than the nuclear ribosomal DNA [14].

The characterization of the mt genomes of *N. oiratianus* and *N. spathiger* also allows a reassessment of the systematic relationships of nematodes within the Trichostrongyloidea using mt proteomic datasets. For many years, there has been considerable debate surrounding the systematics of members within the Trichostrongyloidea (including Haemonchidae, Cooperiidae, Trichostrongylidae, Molineidae and Dictyocaulidae) [47]. Given the controversy, concatenated mt proteomic/genomic datasets might be applied effectively to genetically characterize and compare Trichostrongyloid nematodes. The current work has provided insight into the phylogenetic relationships among Trichostrongyloid nematodes, however, many species of Trichostrongyloid nematodes are not represented. Expanding taxon sampling is therefore necessary for future phylogenetic studies of a wide range of Trichostrongyloidea using mt proteomic/genomic datasets.

## Conclusions

The present study determined the complete mt genome sequences of *N. oiratianus* and *N. spathiger*. The mtDNA data presented here not only provide new mtDNA resources for a better understanding of nematode mt genomics and phylogeny, but also provide novel and useful genetic markers for studying diagnosis, population genetics, and molecular epidemiology of *Nematodirus* spp. in small ruminants.

## Competing interests

The authors declare that they have no competing interests.

## Authors’ contributions

GHZ and GHL conceived and designed the study, and critically revised the manuscript. GHZ, YQJ, WYC, WZ and QQB performed the experiments, analyzed the data and drafted the manuscript. All authors read and approved the final manuscript.

## Supplementary Material

Additional file 1 **Sequences of primers used to amplify PCR fragments from *Nematodirus oiratianus *** **and *N. spathiger*.** Click here for file

Additional file 2 **Phylogenetic relationships of *Nematodirus *** **spp. inferred by maximum parsimony (MP) and maximum likelihood (ML) analyses based on ITS-2 rDNA sequences.***Ostertagia* sp. is used as the outgroup. Posterior probabilities/bootstrap values (in percentage) above 50% from 1,000 pseudo-replicates are shown for the MP (the first value), and ML analyses (the second value). MP analysis was performed using PAUP* 4.0 Beta10 program with default parameters. ML analyses were performed using PhyML 3.0 with the GTR model. Click here for file
