# Supplementary material for: Characterization of the complete mitochondrial genomes of Nematodirus oiratianus and Nematodirus spathiger of small ruminants
Source: Parasit Vectors. 2014 Jul 11;7:319. doi: 10.1186/1756-3305-7-319 (PMC4105107; doi:10.1186/1756-3305-7-319)
Supplement: Additional file 1 — Sequences of primers used to amplify PCR fragments from Nematodirus oiratianus and N. spathiger . [file 1756-3305-7-319-S1.docx]

**Additional file 1.** Sequences of primers used to amplify PCR fragments from *Nematodirus oiratianus* and *N. spathiger*.

| **Fragment** | **Position** | **Primer** | **Sequence (5’ to 3’)** | **Size (bp)** | |
| --- | --- | --- | --- | --- | --- |
|  |  |  |  | ***N. oiratianus*** | ***N. spathiger*** |
| mt1 | *rrn*L-*nad*1 | 39F | TAAATGGCAGTCTTAGCGTGA | 4765 | 4741 |
|  |  | 42R | CCCAATAAATGACGCTCATA |  |  |
| mt2 | *nad*1 | F1 | GATTATTTAGTGACGGTTCAAATCATCCAT | 511 | 510 |
|  |  | R1 | AAACTTAATATATCAAAAAGATATGGCAAA |  |  |
| mt3 | *nad*1-*cox*1 | 5F | TATGAGCGTCATTTATTGGG | 7084 | 7060 |
|  |  | JB4.5 | TAAAGAAAGAACATAATGAAAATG |  |  |
| mt4 | *cox*1-*rrn*L | F2 | CATTTGGTATTATTAGTCAGTCTACTTTAT | 2330 | 2320 |
|  |  | R2 | TATTATTAAAAAAATTTCCGAAGACTTATC |  |  |
