# Supplementary figures and images for: Characterization of the complete mitochondrial genomes of Nematodirus oiratianus and Nematodirus spathiger of small ruminants
Source: Parasit Vectors. 2014 Jul 11;7:319. doi: 10.1186/1756-3305-7-319 (PMC4105107; doi:10.1186/1756-3305-7-319)

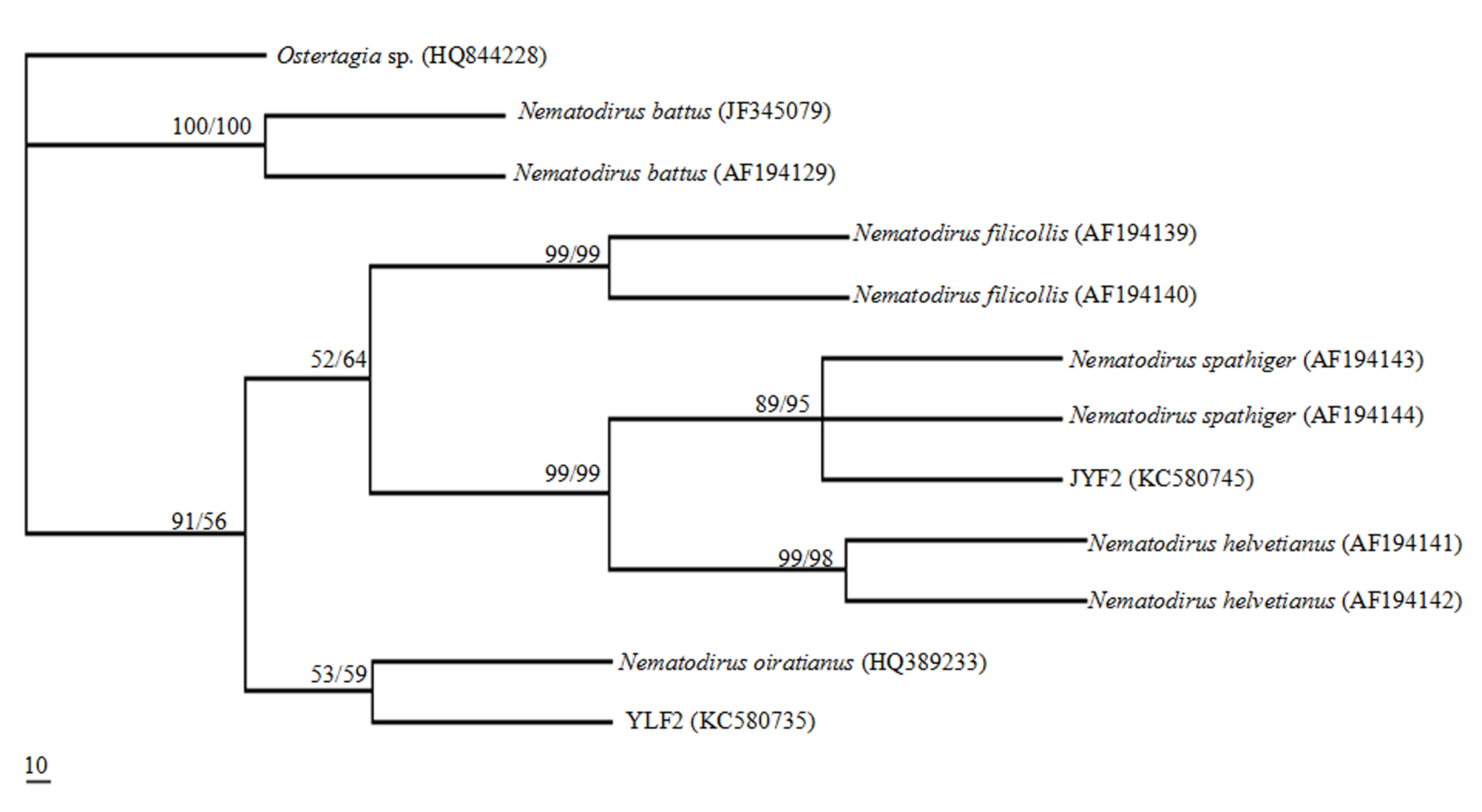

Supplement: Additional file 2 — Phylogenetic relationships of Nematodirus spp. inferred by maximum parsimony (MP) and maximum likelihood (ML) analyses based on ITS-2 rDNA sequences.Ostertagia sp. is used as the outgroup. Posterior probabilities/bootstrap values (in percentage) above 50% from 1,000 pseudo-replicates are shown for the MP (the first value), and ML analyses (the second value). MP analysis was performed using PAUP* 4.0 Beta10 program with default parameters. ML analyses were performed using PhyML 3.0 with the GTR model. [file 1756-3305-7-319-S2.tiff]
